# A Good Compromise: Rapid and Robust Species Proxies for Inventorying Biodiversity Hotspots Using the Terebridae (Gastropoda: Conoidea)

**DOI:** 10.1371/journal.pone.0102160

**Published:** 2014-07-08

**Authors:** Maria Vittoria Modica, Nicolas Puillandre, Magalie Castelin, Yu Zhang, Mandë Holford

**Affiliations:** 1 Department of Biology and Biotechnology “C. Darwin”, Sapienza University of Rome, Roma, Italy; 2 Département Systématique et Evolution, “Systématique, Adaptation et Evolution”, UMR 7138, Muséum National d’Histoire Naturelle, CP 26, Paris, France; 3 Fisheries and Oceans Canada, Pacific Biological Station, Nanaimo, British Columbia, Canada; 4 Ernst & Young, New York, New York, United States of America; 5 Department of Chemistry, Hunter College, City University of New York, New York, New York, United States of America; 6 Sackler Institute for Comparative Genomics, American Museum of Natural History, New York, New York, United States of America; Fordham University, United States of America

## Abstract

Devising a reproducible approach for species delimitation of hyperdiverse groups is an ongoing challenge in evolutionary biology. Speciation processes combine modes of passive and adaptive trait divergence requiring an integrative taxonomy approach to accurately generate robust species hypotheses. However, in light of the rapid decline of diversity on Earth, complete integrative approaches may not be practical in certain species-rich environments. As an alternative, we applied a two-step strategy combining ABGD (Automated Barcode Gap Discovery) and Klee diagrams, to balance speed and accuracy in producing primary species hypotheses (PSHs). Specifically, an ABGD/Klee approach was used for species delimitation in the Terebridae, a neurotoxin-producing marine snail family included in the Conoidea. Delimitation of species boundaries is problematic in the Conoidea, as traditional taxonomic approaches are hampered by the high levels of variation, convergence and morphological plasticity of shell characters. We used ABGD to analyze gaps in the distribution of pairwise distances of 454 *COI* sequences attributed to 87 morphospecies and obtained 98 to 125 Primary Species Hypotheses (PSHs). The PSH partitions were subsequently visualized as a Klee diagram color map, allowing easy detection of the incongruences that were further evaluated individually with two other species delimitation models, General Mixed Yule Coalescent (GMYC) and Poisson Tree Processes (PTP). GMYC and PTP results confirmed the presence of 17 putative cryptic terebrid species in our dataset. The consensus of GMYC, PTP, and ABGD/Klee findings suggest the combination of ABGD and Klee diagrams is an effective approach for rapidly proposing primary species proxies in hyperdiverse groups and a reliable first step for macroscopic biodiversity assessment.

## Introduction

The practice of identifying biological diversity at the species level, referred to as species delimitation, usually consists of first proposing a primary partition of species hypotheses, and then testing these hypotheses. However, when novel taxa are almost completely unknown, such as in hotspot habitats of high diversity as found in recent explorations of the deep-sea [1], [2] or forest canopy [3], a hypothesis-driven approach is not possible as primary species hypotheses (PSHs) are not available for such groups. In high diversity environments, an exploratory DNA based approach, such as DNA barcoding, has been instrumental in producing primary species hypotheses [4]–[6] The current standard for producing and/or testing PSHs is the integration of molecular, and if possible, multi-locus/genomic data, with morphological, ecological, behavioural, geographical characters that are analyzed using multiple criteria such as similarity, phylogeny, and reproduction tested directly or indirectly via gene flow estimations [7]–[10]. The order in which characters and criteria should be applied and which characters are more reliable is debateable [11]–[19]. Additionally, any proposed strategy of species delimitations has to confront two conflicting aims: A) Producing robust species hypotheses and B) Accelerating the pace of species delimitation/description in the context of the growing magnitude of unknown biodiversity and the increasing rate of biodiversity extinction. Integrative taxonomy fulfills the first deliverable of 20^th^ century taxonomy, i.e. it can propose robust species hypotheses, but is not a strategy that can meet the requirements for a rapid survey of species diversity, such as in floral hotspots. The analysis required to obtain robust species delimitations using a fully integrative taxonomy approach can be at times unattainable for a number of reasons such as: (1) technical, e.g. in hyperdiverse groups, characterized by an exceedingly high species number, relatively few available variable genes and inapplicable morphological characters; (2) economical, e.g. lack of funds to obtain and analyze sufficient specimens and characters; or (3) strategic, the need to rapidly assess the diversity of a group or an environment that is potentially threatened. In an effort to address these issues, there has been an increase in species delimitation methods with over 60% being published after 2008 [20]. Carstens and colleagues recently reviewed several species delimitation methods and suggest species delimitations are robust when several delimitation analyses are applied and are congruent [21].

**Figure 1:**
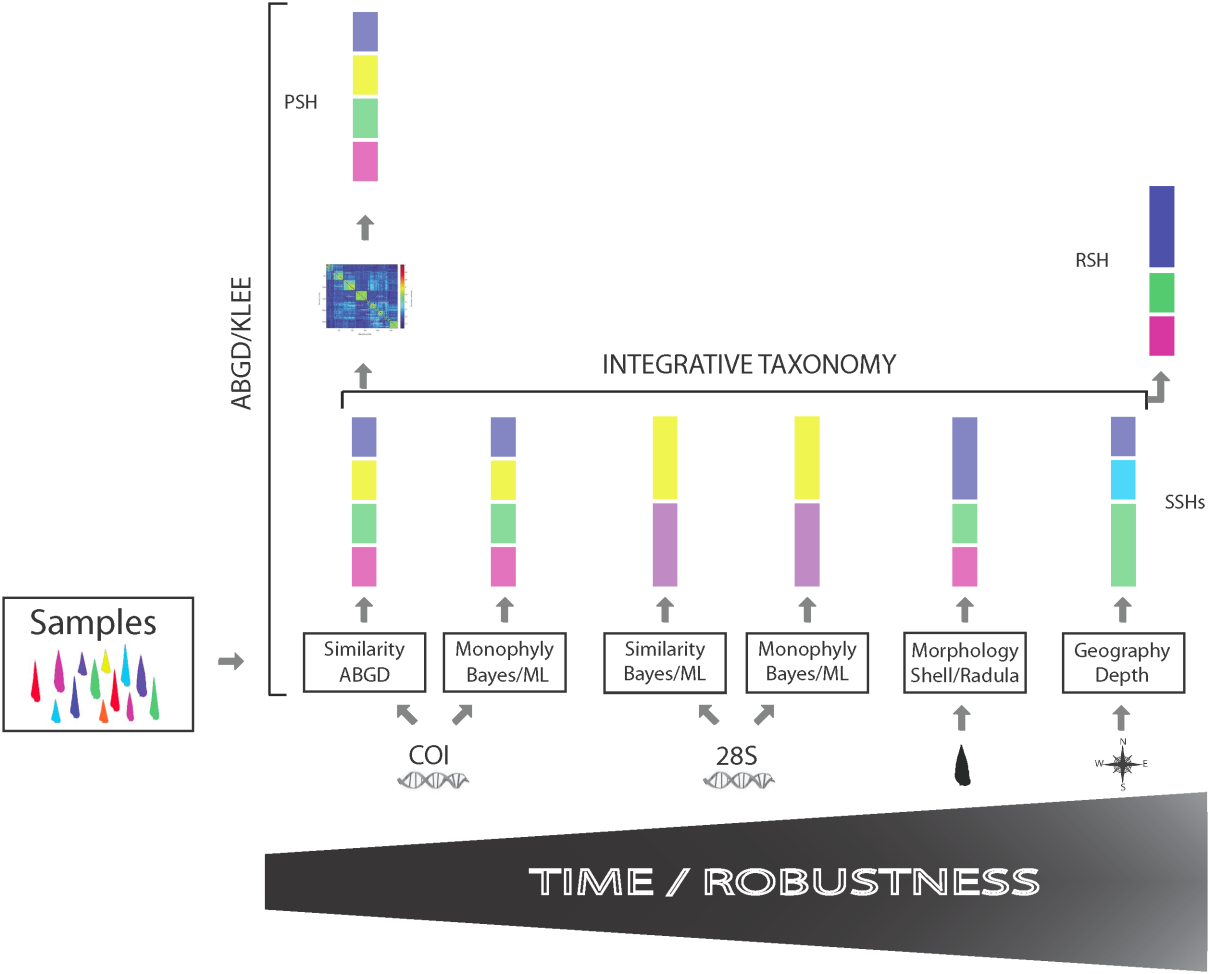
ABGD/Klee species proxy strategy. Species delimitation is shown as a function of time and robustness. ABGD/Klee allows for a fast and relatively accurate first assessment of species diversity. A sampling of biodiverse taxa is first analyzed by bioinformatics species delimitation tool ABGD (Automated Barcode Gap Discovery) using the *COI* gene and visualized by Klee diagrams generated from indicator vectors of *COI* allowing primary species hypotheses (PSHs) to be made. Further analyses using integrative taxonomy in which additional characters (genes, morphology, geography) and criteria (similarity, phylogeny) will generate secondary species hypotheses (SSHs), but this involves a significant increase in time to produce a definitive robust species hypothesis (RSH).

**Figure 2:**
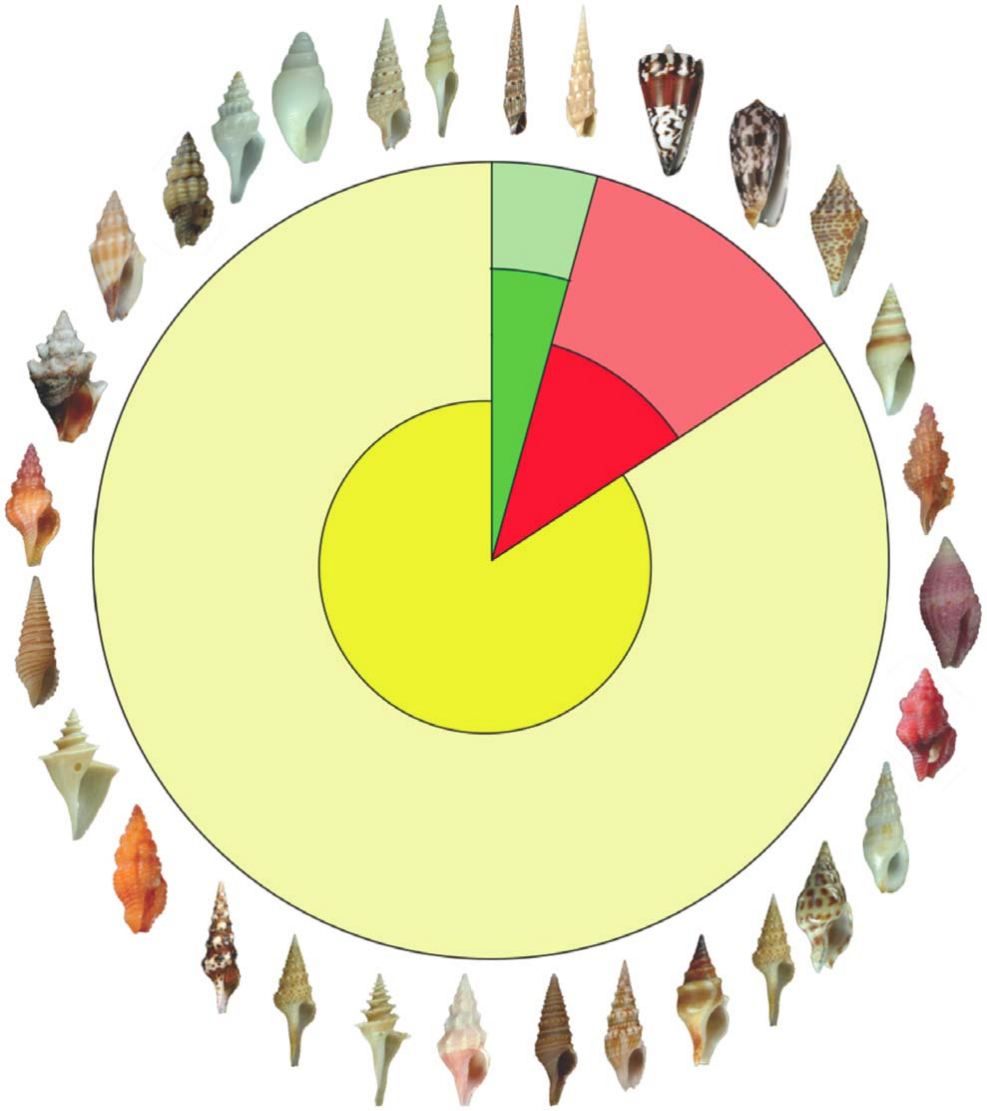
Known and estimated conoidean biodiversity. The three predatory marine mollusk groups of Conoidea are illustrated with representative shells. Conidae (cone snails) in red, Terebridae (auger snails) in green, and the 14 remaining families, referred to as turrids, in yellow. The inner dark colors refer to known diversity and the outer light colors refer to estimated diversity.

With the aim of finding a balance between rapidity and robustness, the two-step strategy presented here uses the cytochrome C oxidase subunit 1 mitochondrial region (*COI*) Barcode fragment to propose Primary Species Hypotheses (PSHs) based on analysis of species delimitation tool ABGD (Automatic Barcode Gap Discovery) [22], combined with Klee diagrams, a graphical mathematical method for effectively visualizing large datasets such as the Terebridae [23], [24] (Fig. 1). The ABGD/Klee strategy addresses four of five criteria of integrative taxonomy as outlined by Padial and colleagues [25]. Namely, ABGD/Klee improves taxonomic work protocol, refines the probabilistic procedures to evaluate character congruence, develops modular software for species delimitation, description, and publishing, and can be considered a semi-automated approach for identification of PSH candidates. To date, a mere 4,500 of the estimated 10,000–20,000 species of the Conoidea, a group of hyperdiverse venomous marine snails that include cone snails (Conidae), auger snails (Terebridae) and turrids, are described [26], [27] (Fig. 2). Increasing the rate of species description for conoideans is crucial for two main reasons: (1) Their potential susceptibility to environmental threats, as many of them are members of coral reef communities; and (2) conoidean venoms are rich in neuropeptides that are important tools for biochemical investigations of neuronal signaling and have relevant pharmacological applications. Conoidea is one of the most promising animal groups for the discovery of novel pharmacologically active neuropeptides, as exemplified by the development of the first drug from a cone snail conopeptide, ziconotide (Prialt), which is used to alleviate chronic pain in HIV and cancer patients [28]. Traditional taxonomic approaches, based mainly on shell characters, are of little value to identify conoidean species [29], [30], and recent DNA-based taxonomic studies demonstrated that the traditional taxonomic framework of conoideans is largely inadequate [27], [31]–[33]. A recent large-scale survey of species diversity in the Turridae revealed that an exploratory approach using ABGD and Klee diagrams was useful to quickly define numerous PSHs, which were confirmed as valid species with additional evidence [27]. In order to validate the ABGD/Klee approach with another group, a similar analysis has been carried out here on the Terebridae. The Terebridae was chosen as it is a well-characterized family of Conoidea that includes ∼350 described species, with an estimated total number of 450 extant species (WORMS–www.marinespecies.org). Recent molecular surveys indicate that most terebrid morphologically defined species are generally congruent with DNA-based clusters [33], which constitutes an exception for the conoideans, making terebrids a good model to test the ability of the ABGD/Klee approach to accurately delimit PSHs.

## Methods

### Ethics statement

Collection permits were provided by the Smithsonian Tropical Research Institute Permit Office (STRI-SPO) and the Panama Aquatic Resources Authority (ARAP) for East Pacific localities and by the Muséum National d’Histoire Naturelle, Paris for all the other localities. Specific locations of collection sites are recorded in Table S1. Our study did not involve endangered or protected species.

### Sample collection

Specimens were collected during several expeditions, mostly in the West and East Pacific (Table S1, Figure 1) and stored in the Malacology Collection of the Muséum National d’Histoire Naturelle (Paris, France).

### Specimen identification

*COI* (cytochrome oxidase C subunit I, *COI*) and 28S rDNA gene sequence data were produced using standard methodologies as detailed in Supplementary Materials (see also Castelin et al. [32] for choice of outgroups and GenBank accession numbers). To briefly describe DNA sequencing and PCR amplification procedures, total genomic DNA was extracted from muscle tissue using NucleoSpin 96 Tissues (Macherey-Nagel). Primers used for *COI* and 28S rDNA (hereafter referred to as 28S) genes were as described in Castelin et al. [32]. PCR reactions were performed in 25 µL final volume, containing approximately 3 ng template DNA, 1.5 mM MgCl2, 0.26 mM of each nucleotide, 0.3 µM of each primer, 5% DMSO and 0.75 U of Taq Polymerase (Qbiogene). PCR amplification products were generated by an initial denaturation step of 4 min at 94°C followed by 35 cycles at 94°C for 40 s, annealing at 50°C for *COI* and 52°C for 28S for 40 s, and by an extension at 72°C for 1 min. All sequenced individuals were examined by Y. Terryn, a taxonomy specialist of the group, and by MH, and were segregated into 87 morphospecies on the basis of shell characters. Specimens of each PSH were attributed to a species name based on the taxonomic literature and on the similarity with identified reference shells available in the Malacology Collection of the MNHN.

### Species delimitation

DNA sequences were aligned with MUSCLE 3.8.31 [34] and accuracy of the alignment was confirmed by eye.

To propose molecular PSHs, 454 *COI* sequences were analyzed using the ABGD method (http://wwwabi.snv.jussieu.fr/public/abgd/abgdweb.html), which tentatively detects for a series of prior thresholds a gap in the pairwise distribution of genetic distances that would eventually correspond to the upper limit of intraspecific distances and lower limit of interspecific distances. A partition of PSHs is given for each prior threshold tested; each PSH of these initial partitions are then recursively tested to eventually detect a second gap in the distribution and propose a recursive partition. The most inclusive (lumper) and the least inclusive (splitter) among ABGD partitions proposed were taken into consideration. To visualize incongruence between these partitions, one sequence of each PSH of the splitter partition was used to build indicator vectors according to Sirovich et al. [23], [24] to produce a Klee diagram. Specimens showing >90% of indicator vector similarity were considered to belong to the same species, and grouped into corresponding PSHs. Support values for monophyletic PSHs in both *COI* and 28S (for a subset of taxa) phylogenies were compared and evaluated. Maximum Likelihood phylogenetic inference (ML) was performed for both genes using RAxML 8.1.8 [35], with a GTR substitution matrix [36] and a Γ-distributed model of among-site rate heterogeneity with four discrete rate categories [37]. Three partitions were defined for the *COI* gene, corresponding to each position of the codon. Accuracy of the results was assessed by bootstrap (1000 replicates) using the rapid bootstrap implemented in RAxML 8.1.8 [38]. Bayesian Analyses (BA) were performed running two parallel analyses in MrBayes 3.1.2 [39], consisting each of eight Metropolis-coupled Markov chains of 50,000,000 generations each with a 10,000-step thinning. The number of chains was set to four, and the chain temperature at 0.02. A GTR substitution model with six substitution categories and a Γ-distributed rate variation across sites approximated in four discrete categories was applied for each gene (and each of the three partitions of the *COI* gene). Convergence of each analysis was evaluated using Tracer 1.4.1 [40], and analyses were terminated when ESS values were all superior to 200. A consensus tree was then calculated after omitting the first 25% trees as burn-in.

To evaluate the PSHs proposed with the ABGD/Klee approach, ABGD/Klee species delimitations were compared with clustering obtained from two different species delimitation tools, General Mixed Yule Coalescent (GMYC) and the Poisson Tree Processes (PTP). Unlike ABGD/Klee, GMYC and PTP use a previously generated phylogenetic hypothesis to delimitate species boundaries. GMYC infers species boundaries using the differences of the branching rates in an ultrametric phylogenetic tree to discriminate between inter and intraspecific branching events. In the single-threshold version of the method the switch from speciation to coalescence is supposed to be unique [41], while in the multiple thresholds version the initial species partition can be recursively re-analyzed to further split or join species [42]. Here we generated an ultrametric tree in BEAST 1.7.5 [43], using a site-specific GTR substitution matrix [36] and a Γ-distributed model of among-site rate heterogeneity with four discrete rate categories [37]. Relative divergence times were estimated running four relaxed lognormal clock analyses with a coalescent prior and a constant population size, that according to Monaghan et al. [42] are the best-fitting parameters to be used in GMYC analyses. Convergence of each analysis was evaluated in Tracer 1.4.1 [40], and analyses were interrupted when ESS values exceeded 200. After excluding the first 25% trees as burn-in, a consensus tree was calculated. The consensus tree was then used to infer species delimitation with the GMYC method, using both the single and the multiple thresholds methods, with the package SPLITS in R [44], [45]. On the contrary, PTP does not require an ultrametric tree, as the transition point between intra- and inter-specific branching rates is identified using directly the number of nucleotide substitution [46]. PTP incorporates the number of substitutions in the model of speciation and assumes that the probability that a substitution gives rise to a speciation event follows a Poisson distribution. The branch lengths of the input tree are supposed to be generated by two independent classes of Poisson events, one corresponding to speciation and the other to coalescence. The ML phylogeny obtained with RAxML as the input tree was used as described previously, and PTP analysis was run from Python using the ETE (Python Environment for Tree Exploration) package [47] for tree manipulation and visualization.

## Results

A total of 454 specimens of Terebridae were sequenced for a 658-bp fragment of the *COI* gene, while a portion of the *28S* rDNA ranging from 696 to 742 bp was sequenced in a subset of 195 specimens and used to build a 758-bp alignment (Data S1 and S2). The *COI* alignment was analyzed with ABGD to propose partitions with variable numbers of PSHs, depending on the prior threshold and initial or recursive analyses. The more inclusive (lumper) partition provided by ABGD included 98 clusters, and the least inclusive (splitter) partition contained 125 clusters. Based on the *COI* gene only, GMYC and PTP analyses contained a variable number of clusters, mostly overlapping with ABGD: 110 in the GMYC single threshold, 130 in the GMYC multiple threshold and 112 in the PTP (Fig. S1–S3). Sixty-three PSHs are found identical in the five partitions. If the partitions obtained with the GMYC multiple threshold method are excluded, the number of identical PSHs raises to 83.

**Figure 3:**
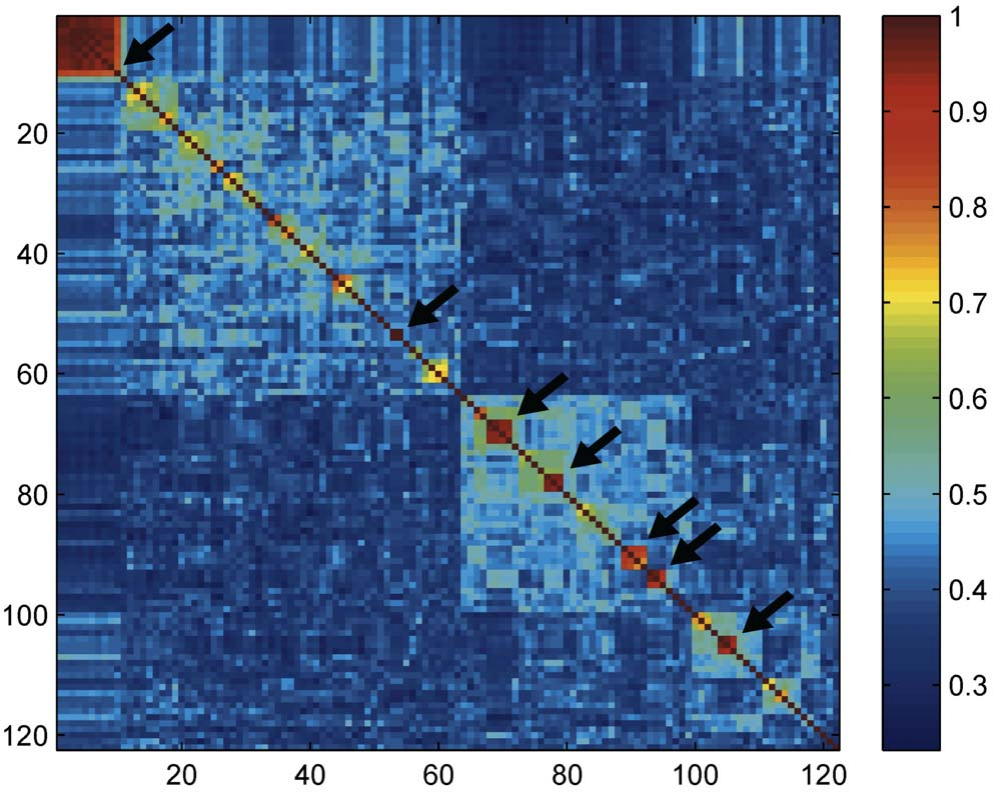
Terebridae Klee diagram. Klee diagram for the *COI* gene showing the correlation amongst indicator vectors for the less inclusive (splitter) dataset obtained with the ABGD method and including 125 PSHs. Color gradation in red indicates high correlation values. Arrows indicate the conflicting PSHs between the more inclusive and the less inclusive partitions discussed in the text and listed in Table S1.

Eighty-seven morphospecies were identified from the analyzed terebrid dataset, representing about 25% of the known diversity of the family, which corresponds to 12 genera as defined in Terryn, 2007 [48], and further classified in Castelin et al. [32]. Sixty-nine morphospecies were linked to a unique species name, one is similar to *Terebra variegata* and 17 were assigned only to a genus name (designated by “sp.”) (Table S1). Eight morphospecies were split in two or three PSH in both the lumper and the splitter ABGD partition, namely: *Triplostephanus fenestratus* (PSHs 16 and 31), *T. triseriatus* (PSHs 20 and 81), *Clathroterebra fortunei* (PSHs 17 and 93), *Hastula strigilata* (PSH 26, 28 and 34), *Hastulopsis pertusa* (PSHs 15 and 49), *Strioterebrum plumbeum* (PSHs 47 and 63), *Terebra succincta* (PSHs 2 and 13) and *T. textilis* (PSHs 4, 79 and 80). The same pattern is observed in the results from other species delimitation methods (GMYC single, GMYC multiple and PTP) (Table S1). In most cases, the two or three PSHs sharing a single morphospecies name were not closely related, and Klee diagrams highlighted the low correlation values between them (Fig. 3). Most PSHs identified are monophyletic with high support values in the *COI* phylogeny.

Additionally, ten PSHs defined in the lumper partition were split in several PSH in the splitter partitions. Incongruence between the lumper and splitter ABGD partitions can be easily visualized and evaluated when sequence data corresponding to the splitter partition are transformed in indicator vectors and used to build a Klee diagram with the indicator vector method [23], [24] (Fig. 3).

**Figure 4:**
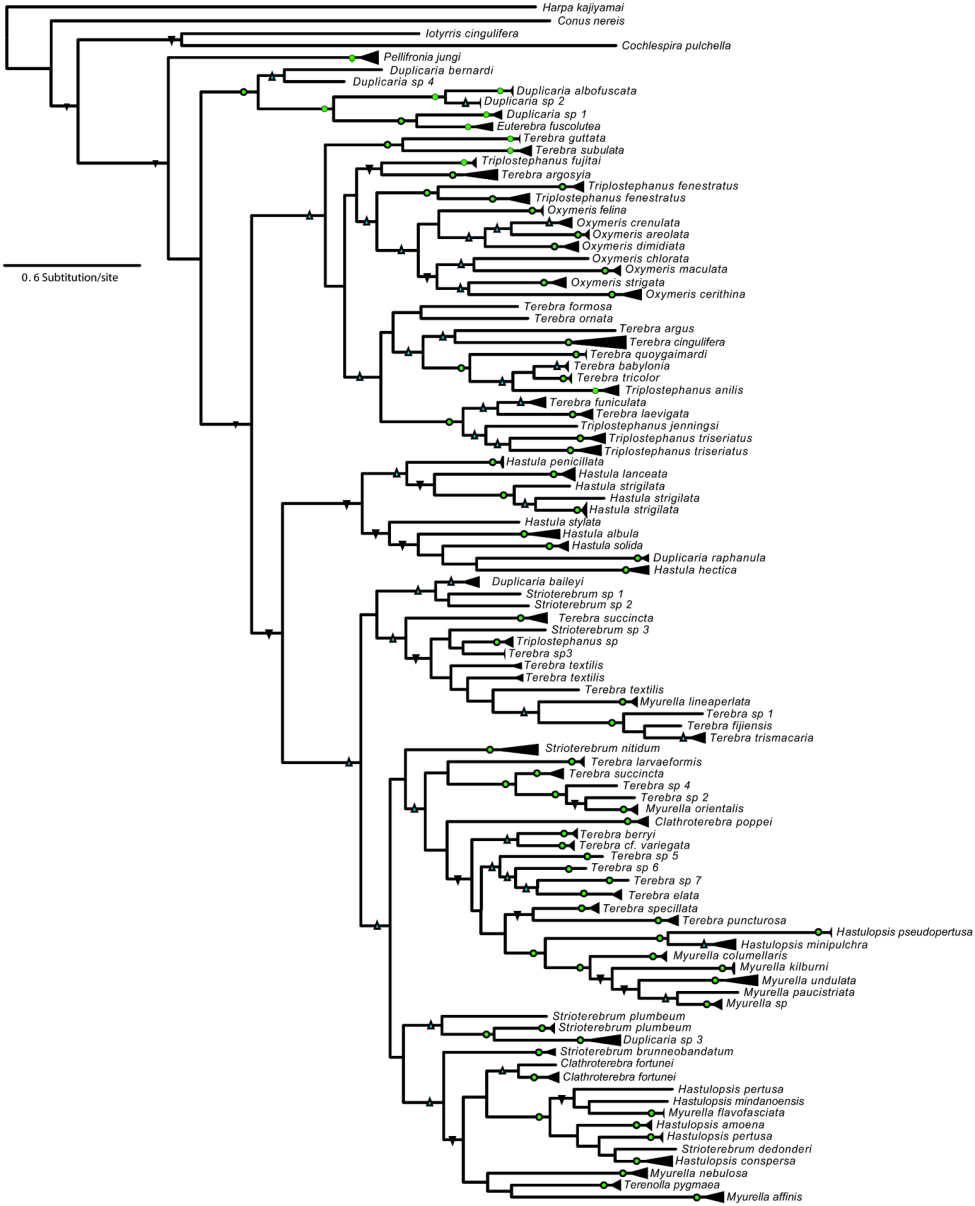
Terebridae COI phylogeny. Bayesian phylogenetic tree estimated with the *COI* gene alignment. Clades including several specimens identified as a single morphospecies are compressed in triangles. Green circles indicate PP = 100; Blue upward triangles indicate PP>80; Black downward triangles indicate PP>50.

**Figure 5:**
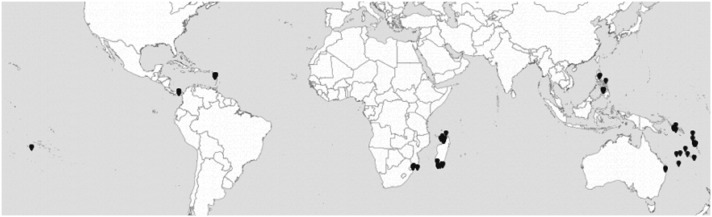
Geographical distribution of Terebridae specimens analyzed.

For five groups of PSHs, 13a–b, 24a–d, 71a–c, 81a–c and 98a–c, the single PSHs identified in the splitter partition are barely distinguishable in the Klee diagram due to the high correlation (>90%) between indicator vectors of the PSHs in each group (Fig. 3). This observation is confirmed by the low support values obtained in the *COI* phylogenies for the PSHs groups in the splitter partition vs. the lumper partition (Fig. 4 & Table S1). Additionally, *28S* gene sequences were paraphyletic between members of each splitter PSH. On the basis of these results 13a–b, 24a–d, 71a–c, 81a–c and 98a–c PSHs were rejected and not considered candidate species. However, three groups of PSHs from the same partition as those rejected, 3a–d, 12a–b, 30a–b, were clearly recognized in the Klee diagram (Fig. 3). These splitter PSH groups are mostly monophyletic in the *COI* phylogeny, with support values comparable or only slightly lower than the lumper partition. Additionally, results obtained from the GMYC and PTP are congruent and support the splitting of partitions. For 3a–d, 12a–b, 30a–b PSH groups, 28S gene results either confirmed the monophyly of the group (e.g. for 30a–b) or were inconclusive. These results substantially reflect a geographical differentiation. Specifically, *Terebra cingulifera* (PSH3) appears split in four species, 3a from Philippines and Solomon Islands, 3d from Philippines, 3b and c from Vanuatu (Fig. 5). PSHs 12a and 12b were identified as the single morphospecies *Myurella undulata*, respectively from Vanuatu and West Africa. The same pattern is observed in *Strioterebrum nitidum,* with PSH 30a from Vanuatu and 30b from East Africa. As a result, 3a–d, 12a–b, 30a–b PSHs, referring to *T. cingulifera, M. undulata, and S. nitidum* were accepted as sound candidate species.

A more complex pattern was retrieved for one of the two cluster morphologically identified as *Triplostephanus fenestratus* (PSH 16) and *Duplicaria* sp. 3 (PSH 33). PSHs 16 and 33 were split respectively in three (PSHs 16a–c) and ten (PSHs 33a–j) partitions in the ABGD splitter analysis. Inspection of the Klee diagram for PSH 16 and 33 clearly shows that correlation values of indicator vectors are lower than 90% only between two clusters internal to each PSH (Fig. 3). In other words, the Klee diagram only supports a split between PSHs 16a–b and 16c, respectively from Philippines and Madagascar) and between PSHs 33a and 33b–j, respectively from Vanuatu and Madagascar (Fig. 5). This result, although not congruent with ABGD analyses, is supported by GMYC analyses and, in case of PSH 33, by PTP analysis as well. PSHs 16a–b and 16c and 33a and 33b–j were thus accepted as candidate species.

In summary a partition of 104 Primary Species Hypotheses are proposed that are congruent based on different characters (*COI*, 28S), criteria (similarity, phylogeny) and species delimitation methods (ABGD/Klee, GMYC, PTP).

## Discussion

A two-step species delimitation strategy of ABGD and Klee diagrams was used to propose 104 primary species hypotheses for the conoidean family Terebridae. Our results reinforce that the ABGD/Klee strategy is both fast and robust. The majority of PSHs proposed by ABGD/Klee were confirmed by additional evidence, *28S* gene/morphological variability, and species delimitations methods GMYC and PTP, suggesting that a large number of PSHs obtained by ABGD/Klee would be validated by a more comprehensive integrative approach. Congruence across results obtained with different methods is critical to strengthen confidence in proposed species delimitation hypothesis [21]. For the Terebridae dataset used, 17 cryptic species were identified based on congruence of ABCD/Klee, GMYC, and PTP analyses. Except for an apparent overestimation of PSHs in GMYC multiple threshold analysis, general agreement was observed in proposed terebrid species partitioning. Overestimation in species number is a common issue when using a multiple threshold method [49], especially when dealing with species with strong intra-specific genetic structure, due to features such as limited dispersal abilities [50].

While some of the proposed terebrid taxonomic issues presented cannot be resolved without a full integrative taxonomy approach, ABGD/Klee has provided a solid foundation for further investigation. In a number of cases, e.g. in *T. textilis*, (PSHs 79 and 80), *S. plumbeum* (PSHs 47 and 63), *H. pertusa* (PSHs 26, 28 and 34), *T. triseriatus* (PSHs 20 and 81), *T. fenestratus* (SSHs 16 and 31) and *T. cingulifera* (PSHs 3b and 3c), the proposed pairs or triplets of PSHs were collected in at least one common area, and are considered sympatric (Table S1). In such cases the phylogeographic pattern observed strongly supports the results obtained with our approach. The observed levels of genetic differentiation indicate that these ABGD/Klee PSHs correspond to valid species, with a remarkable extent of morphological convergence of their shell features.

In other instances, the identification of two or more PSHs in single morphospecies of our sample correlated with a disjunct geographic distribution, e.g. in *T. succincta* (PSHs 2 and 13), *C. fortunei* (PSHs 17 and 93), *T. textilis* (PSHs 4, 79 and 80), *T. fenestratus* (PSHs 16ab and 16c), *M. undulata* (PSHs 12a and 12b) and *T. cingulifera* (PSHs 3a, 3b, 3c and 3d) (Fig. 5). For these putative allopatric species pairs, a more complete integrative approach taking into account evidence such as dispersal abilities is needed to rule out the possibility that genetic differentiation is due to an intraspecific geographic structure for PSH pairs. In disjoint populations, reduced dispersal abilities are generally linked to higher levels of interpopulation genetic divergence [51]. In marine environment, dispersal ability of benthic organisms is frequently influenced by the duration of their larval stage. This can be extremely variable, even in closely related species, ranging from remarkably long (species with teleplanic planktotrophic larvae), to short (species with lecitotrophic pelagic larvae), or even absent (species with intracapsular development or brooding) [52], [53]. In Caenogastropoda, the mode of larval development can be inferred from the protoconch morphology and has been shown to exert a remarkable influence on microevolutionary processes [53], [54].

Remarkably, there are no cases in which two morphological distinct species are joined in a single PSH using ABGD/Klee approach, suggesting that the use of morphological characters in Terebridae is not likely to lead to alpha errors in biodiversity estimate (e.g. overestimation of the number of species), due to a general lack of informativeness of shell characters.

For the Conoidea, DNA-based taxonomy has frequently resulted in the discovery of new species [31]. More specifically, for the hyperdiverse family Turridae, more than half of the delimited species were not congruent with the morphospecies hypotheses [27]. In that case, the ABGD/Klee strategy coupled with GMYC allowed the identification of 87 species, more than doubling the number species for the genus *Gemmula* alone. In contrast, the number of new candidate species identified for terebrids via the ABGD/Klee approach is roughly 4% of the 350 total number of recognized species. This finding is in agreement with the high congruence generally observed between molecular–based species delimitation and morphospecies hypothesis for the Terebridae [32]. In the terebrid and turrid families of the Conoidea, the ABGD/Klee approach, and more generally, a single gene approach, was successful in defining PSHs, validating this approach for hyperdiverse marine mollusks and other biodiverse organisms. Additionally, as ABGD/Klee is based on a single COI gene analysis it requires less than a few minutes of computation time to analyze relatively large datasets such as the 400–1,000 sequences of conoidean terebrids or turrids. Differently from PTP analysis, which is also relatively fast, ABGD/Klee approach only relies on sequence similarity thresholds. This characteristic makes ABGD/Klee more suitable for hyperdiverse taxa, where robust single gene phylogenies are difficult to obtain and hamper the accurateness of species delimitation in tree-based methods [21]. Another difference is that PTP may overestimate the number of species when taxon sampling is uneven between species [46], a common issue especially in hyperdiverse groups.

Admittedly, the ABGD/Klee strategy may define some PSHs that could be invalidated by a comprehensive total evidence analysis, but for biodiversity hotspots, a tactical approach such as ABGD/Klee is satisfactory, as it represents a good compromise between rapidity and robustness. In instances such as, biodiversity inventories of threatened environments, species richness estimations, and metabarcoding of soil or gut contents, especially in the emergency imposed by the context of the recent increase in the extinction rates, the application of ABGD/Klee would produce stable proxies of species hypotheses in order to advance scientific investigations. In biomedically relevant groups such as the conoideans, time-efficient species delimitation is a fundamental prerequisite for drug discovery [55]. Plurality of characters and methods is important for deciphering temporal order of evolving traits, and relying on a single trait is not ideal, but every race has a starting line, ABGD in combination with Klee diagrams is a robust starting line for species delimitation in hyperdiverse taxa.

## Supporting Information

Figure S1 **Results of GMYC single threshold species delimitation on** ***COI*** **alignment.**
(PDF)

Figure S2 **Results of GMYC multiple thresholds species delimitation on** ***COI*** **alignment.**
(PDF)

Figure S3 **Results of PTP species delimitation on** ***COI*** **alignment.**
(PDF)

Table S1 **List of Terebridae specimens analyzed.** Table indicates morphospecies identification and collection data, together with PSH assignment (ABGD lumper and splitter partitions, GMYC single and multiple thresholds and PTP), statistical support (Bootstraps and Posterior Probabilities) for both *COI* and 28S loci for each defined PSH and Klee results.
(XLSX)

Data S1 **Original alignment of** ***COI*** **gene sequences.**
(FASTA)

Data S2 **Original alignment of 28S rDNA gene sequences.**
(FASTA)
