# Supplementary figures and images for: A Good Compromise: Rapid and Robust Species Proxies for Inventorying Biodiversity Hotspots Using the Terebridae (Gastropoda: Conoidea)
Source: PLoS One. 2014 Jul 8;9(7):e102160. doi: 10.1371/journal.pone.0102160 (PMC4086986; doi:10.1371/journal.pone.0102160)

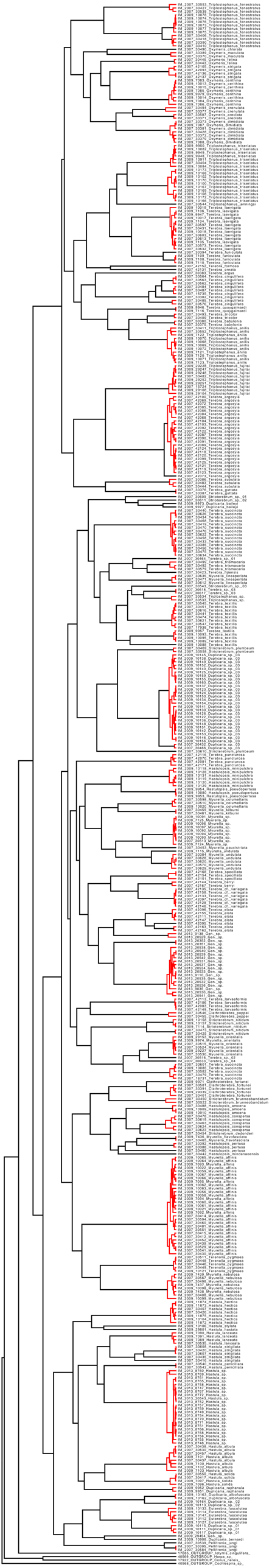

Supplement: Figure S1 — Results of GMYC single threshold species delimitation on COI alignment. (PDF) [file pone.0102160.s001.pdf]

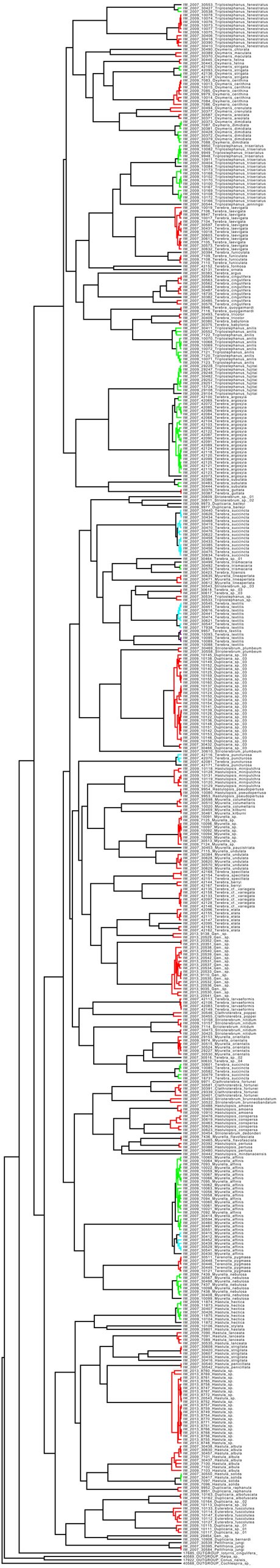

Supplement: Figure S2 — Results of GMYC multiple thresholds species delimitation on COI alignment. (PDF) [file pone.0102160.s002.pdf]

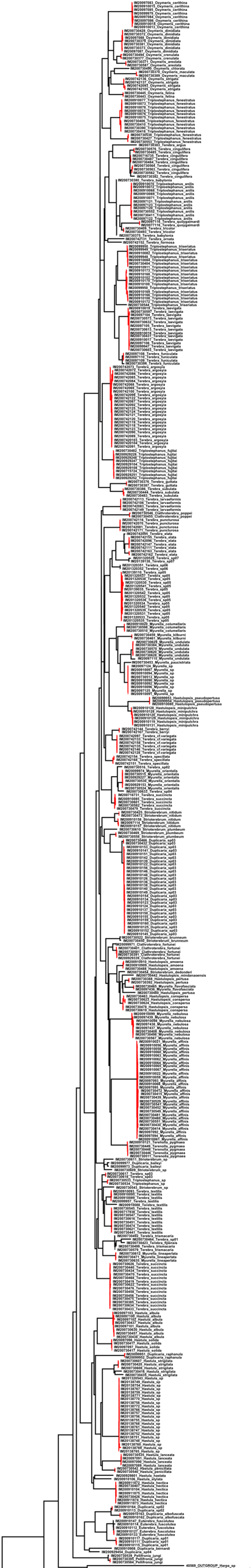

Supplement: Figure S3 — Results of PTP species delimitation on COI alignment. (PDF) [file pone.0102160.s003.pdf]
